# Machine Learning-Based Prediction of Distant Recurrence in Invasive Breast Carcinoma Using Clinicopathological Data: A Cross-Institutional Study

**DOI:** 10.3390/cancers15153960

**Published:** 2023-08-03

**Authors:** Shrey S. Sukhadia, Kristen E. Muller, Adrienne A. Workman, Shivashankar H. Nagaraj

**Affiliations:** 1 Centre for Genomics and Personalised Health, Queensland University of Technology, Brisbane, QLD 4059, Australia; 2 Department of Pathology and Laboratory Medicine, Dartmouth Hitchcock Medical Center, Lebanon, NH 03766, USA; kristen.e.muller@hitchcock.org (K.E.M.); adrienne.a.workman@hitchcock.org (A.A.W.)

**Keywords:** radiogenomics, invasive breast carcinoma, machine learning, neoadjuvant therapy

## Abstract

**Simple Summary:**

Breast cancer is a diverse disease with varying prognoses, even within the same subtype. Approximately 30% of breast cancer patients experience distant organ recurrence, known as metastasis, after treatment. The evaluation of breast tumors and surrounding lymph nodes occurs before and after neoadjuvant therapy, which aims to shrink the tumor before surgery. Following resection, residual tumor cells may remain in the breast tissue, lymph nodes, or other areas, necessitating adjuvant therapy. Typically, a follow-up visit is scheduled a year or more after adjuvant therapy, during which metastasis may be detected. By utilizing machine learning techniques, metastasis can be predicted earlier in a clinical setting, allowing for tailored surveillance and treatment strategies. This has the potential to significantly enhance the quality of life for breast cancer patients.

**Abstract:**

Breast cancer is the most common type of cancer worldwide. Alarmingly, approximately 30% of breast cancer cases result in disease recurrence at distant organs after treatment. Distant recurrence is more common in some subtypes such as invasive breast carcinoma (IBC). While clinicians have utilized several clinicopathological measurements to predict distant recurrences in IBC, no studies have predicted distant recurrences by combining clinicopathological evaluations of IBC tumors pre- and post-therapy with machine learning (ML) models. The goal of our study was to determine whether classification-based ML techniques could predict distant recurrences in IBC patients using key clinicopathological measurements, including pathological staging of the tumor and surrounding lymph nodes assessed both pre- and post-neoadjuvant therapy, response to therapy via standard-of-care imaging, and binary status of adjuvant therapy administered to patients. We trained and tested four clinicopathological ML models using a dataset (144 and 17 patients for training and testing, respectively) from Duke University and validated the best-performing model using an external dataset (8 patients) from Dartmouth Hitchcock Medical Center. The random forest model performed better than the C-support vector classifier, multilayer perceptron, and logistic regression models, yielding AUC values of 1.0 in the testing set and 0.75 in the validation set (*p* < 0.002) across both institutions, thereby demonstrating the cross-institutional portability and validity of ML models in the field of clinical research in cancer. The top-ranking clinicopathological measurement impacting the prediction of distant recurrences in IBC were identified to be tumor response to neoadjuvant therapy as evaluated via SOC imaging and pathology, which included tumor as well as node staging.

## 1. Introduction

Breast cancer—which has surpassed lung cancer as the most common type of cancer worldwide—accounts for nearly one-third of cancers in women [1,2]. Despite considerable advances in detection and treatment, nearly 30% of breast cancer patients will develop distant recurrences (i.e., metastasis), often years after diagnosis and treatment of the primary tumor [3]. Although clinicopathological features and molecular biomarkers have been used previously to guide therapeutic decisions in breast cancer clinical workflows [4], forecast outcomes [5,6,7], and prediction of distant metastases [8,9,10], long-term survival rates and prognoses vary widely, even within the same histologic and molecular subtype [11,12]. Thus, the high degree of heterogeneity both within and between tumors, combined with myriad other factors that affect the evolution of breast cancer within each individual, presents substantial challenges in treatment and clinical management [13].

Neoadjuvant therapy (i.e., any therapy that precedes surgery) has been used widely in breast cancer to improve outcomes by downstaging inoperable primary tumors to facilitate tumor resection [14,15]. Tumor response to neoadjuvant therapy is evaluated using standard-of-care (SOC) radiological imaging techniques such as MRI, PET-CT, or ultrasound [16,17] and/or biopsies of tumor tissue or surrounding lymph nodes [18], and can be a valuable predictor of survival after therapy [15]. Notably, a complete pathologic response with no residual tumor is indicative of an excellent prognosis in patients with HER2-positive and triple-negative tumors [19,20]. After surgical removal of the primary tumor, adjuvant therapy can be administered to eliminate any residual tumor in breast tissues or possibly in any other part of the body [21,22].

Distant recurrences post adjuvant therapy are common in patients with certain subtypes of breast cancer, such as invasive breast carcinoma (IBC) [23]. Breast cancer most frequently metastasizes to the bone, lung, liver, brain, and distant lymph nodes, of which bone is the most prominent metastatic site [23,24]. As metastatic disease—the primary reason for breast cancer-related deaths—is known to have poor outcomes and is generally considered to be incurable, predicting distant recurrences is a crucial aspect of personalized monitoring and treatment strategies [3]. While breast cancer subtype can be predictive of the preferred site of distant metastasis [9], accurately predicting the risk of metastasis is more complex. Although OncotypeDX (ODX)—a 21-gene molecular screening assay—is widely used in clinical practice to predict the distant recurrence and benefits from adjuvant chemotherapy for patients with breast cancer, its utility is limited as the ODX recurrence score is not consistent across all ages and relevant only in women with estrogen receptor (ER)-positive breast cancer, which limits its scope [25,26,27]. The benefits of adjuvant chemotherapy vary depending on the range of the ODX recurrence score when combined with patient age, for which the ideal conditions are women ≤ 50 years with an ODX recurrence score range of 16–25 [28], therefore limiting the patient group for ODX-based tumor recurrence evaluation to younger to mid-aged women only.

Machine learning (ML) has become an increasingly popular tool for making objective clinical predictions and has been used to predict a wide variety of clinical outcomes using biological or clinical input data [29,30,31,32,33,34,35]. Metastasis-risk calculators (i.e., “nomograms”), which are based on logistic regression models, have been known to predict synchronous metastases in breast cancer based on the clinicopathological profile (i.e., tumor size, nodal status, and estrogen and progesterone receptor status); however, this methodology does not involve the usage of adjuvant (i.e., second-line) therapy usually given to patients that may not have a complete response to neoadjuvant therapy (Boutros et al. 2015) [36]. A mechanistic model developed by Nicolò et al. [37] predicts time to metastases in patients with early-stage breast cancer using several clinicopathological diagnoses, but it neither considers patients that received adjuvant therapy nor conducts validation of the model using an external cohort. Another study used various clinicopathological features, including serum HER2 levels, to predict metastases; however, low AUC values in the testing set (i.e., AUC < 0.80) in conjunction with lack of validation of the model using an independent (or external) validation set highlights the low clinical utility of the model [38].

Another factor that affects the clinical utility of predictive ML models in breast cancer is the availability and accessibility of code. For example, the aforementioned studies do not share the complete code (i.e., a package of programming scripts) used to train and test the respective ML models, thereby placing the burden on other researchers to unpack the black box of ML programming and/or attempt to translate the methodology into code that can be applied to their own research. Moreover, these studies do not provide the final (i.e., tested) model pickle files (i.e., model.pkl) that a researcher could potentially use to validate their model using their own (i.e., external) dataset. ML models do not contain sensitive personal health information; rather, they contain mathematical equations that are produced from the training conducted on deidentified patient data. These equations consist of correlation weights or the feature importance scores of features used to train the model, both of which are an indication of each feature’s individual contribution towards a collective prediction of a particular outcome [39]. These weights or importance scores must be tested using the test dataset, independently of the training set, to arrive at a model worthy of validation.

Further, sharing ML models across institutions could enable meta-learning, potentially yielding superiorly performing models [40]. Our recently released version (v3.2) of the ML software program ImaGene [41] exposes its codebase and automates ML operations for an expanded list of classification-based models such as random forest (RF), C-support vector classifier (SVC), multilayer perceptron (MLP), and logistic regression (LogitR) (i.e., in addition to the previously existing regression-based models). ImaGene also offers various customizable parameters to conduct multiple simultaneous ML experiments and provides result metrics such as R-square, RMSE:Stdev ratio, and AUC, the majority of which are plotted as graphs and included in resulting reports. These reports, along with the subordinate tabular output files, aid users in tracking feature-level performances for the respective models.

Early predictions of the risk of metastases in breast cancer patients post adjuvant-therapy and pre-follow-up (for follow-ups of greater than a year) in clinics using an ML model could allow early clinical intervention and guide surveillance and treatment, which could ultimately improve the quality of life for the respective patients. Furthermore, increasing the availability and accessibility of such an ML model across various hospital sites could facilitate the model’s validation and make it clinically viable sitewide. Thus, the objective of our study was twofold: (i) to create a novel, high-fidelity ML model to predict the risk of metastasis in IBC patients using clinicopathologic measurements pre- and post-neoadjuvant therapy, such as clinical tumor [T] and node [N] staging pre-neoadjuvant therapy, response evaluation (via SOC imaging, and T and N staging) post-neoadjuvant therapy, and administration status (‘Yes’ or ‘No’) and type of adjuvant therapy (i.e., chemotherapy or Anti-Neu HER2) obtained from one hospital site (i.e., Duke University Hospital [DUH]), and (ii) to validate that model externally (i.e., Darthmouth-Hitchcock Medical Center [DHMC]). To accomplish this, we used ImaGene to train and test four classification-based ML/AI models (i.e., RF, SVC, MLP, and LogitR) using the DUH cohort and validate the best-performing model using the DHMC cohort. To the best of our knowledge, this is the first study to test and validate ML models for the prediction of distant recurrences using clinicopathological profiles across various therapies in IBC patient cohorts from two different medical institutions.

## 2. Materials and Methods

The present study used clinicopathological data from a retrospective study of 900 IBC patients at DUH, available via the TCIA portal [42,43]. Of these, 312 patients received neoadjuvant therapies such as chemotherapy, radiation, endocrine hormone-based, or anti-neu/HER2 therapy. Responses to therapy were evaluated using imaging and pathological (i.e., T and N) staging. Additionally, 304 of these patients received adjuvant therapies (i.e., chemo-, radiation-, hormone-, or anti-Neu/HER2-based therapies) depending on their responses to neoadjuvant therapy. As our focus was on patients who received neoadjuvant chemotherapy and had either a partial or a complete response, patient entries pertaining to ungraded tumor responses to therapies or those labeled as ‘Not applicable (NA)’ for any of the clinicopathological features were excluded from further analyses, yielding 161 patient entries.

**Figure 1:**
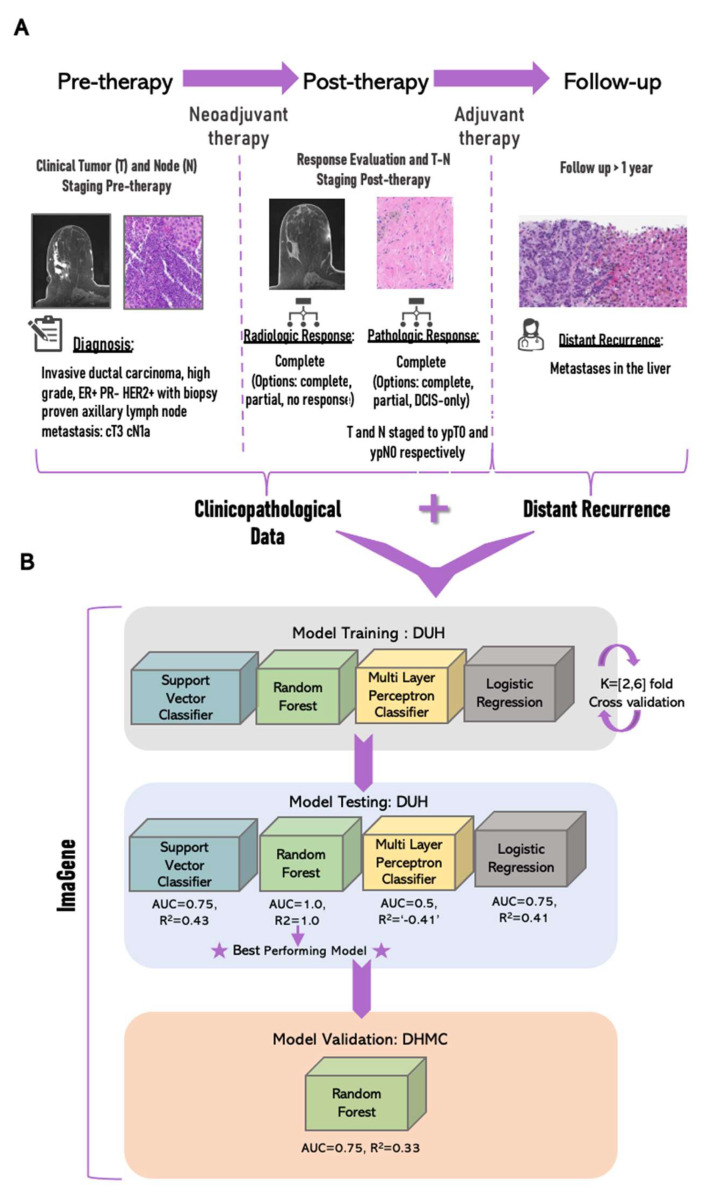
Modeling clinicopathological data to predict distant recurrences in IBC, with clinicopathological workflow for invasive breast cancer (IBC) from initial diagnosis to follow-up represented in (**A**) followed by training, testing, and validation of machine learning (ML) models for the prediction of distant recurrences recorded post-follow-up in IBC using clinicopathological data recorded pre- and post-therapy and pre-follow-up represented in (**B**). The area under the receiver operating curve (AUROC/AUC) and R^2^ values are mentioned for each model, where a negative R^2^ value and AUC = 0.5 indicate model failure. DUH: Duke University Hospital; DHMC: Dartmouth Hitchcock Medical Center.

Specifically, the following eight clinicopathological features (Figure 1A) were considered: (a) tumor stage or size (T0–T4, i.e., in the numerical format: [0,4]) pre-neoadjuvant therapy, (b) staging of lymph nodes (N0–N3, i.e., [0,3]) pre-neoadjuvant therapy, (c) clinical response to neoadjuvant therapy as evaluated using radiological images (i.e., complete response (i.e., ‘1’), incomplete response (i.e., ‘2’), or assessment unavailable (i.e., ‘3’), (d) pathologic response to neoadjuvant therapy (i.e., complete: ‘1′, incomplete: ’2′, or DCIS-only remaining: ‘3’), (e) pathologic response to neoadjuvant therapy as evaluated using pathology stage “T” tumor size (T0–T4, i.e., [0–4]), (f) pathologic response to neoadjuvant therapy evaluated using node status “N” (N0–N3, i.e., [0,3]), (g) status of adjuvant chemotherapy administered (i.e., yes: ‘1’ or no: ‘0’), and (h) status of adjuvant anti-HER2/Neu therapy administered (i.e., yes: ‘1’ or no: ‘0’). Patients with no response to neoadjuvant chemotherapy were excluded from this study, as this patient population has a poor prognosis and a higher risk of recurrence [44]. Model training was conducted using ImaGene software [41].

The 161 patient entries were split further into training and testing sets at a 90:10 ratio, in which 90% of entries were used for training (i.e., ntrain = 144) and 10% for testing (ntest = 17) four classification-based ML models (i.e., RF, SVC, MLP, and LogitR) for binary predictions of distant recurrences (i.e., yes or no). For logistic regression, the train:test ratio was modified to 85:15 to achieve optimal performance. Experiments for the four ML models were performed using ImaGene [41], which yielded operational reports and supporting text files to aid in the interpretation of the results, thereby facilitating the selection of the best-performing model for validation (Figure 1B).

### 2.1. Model Development and Testing Using ImaGene

**Figure 2:**
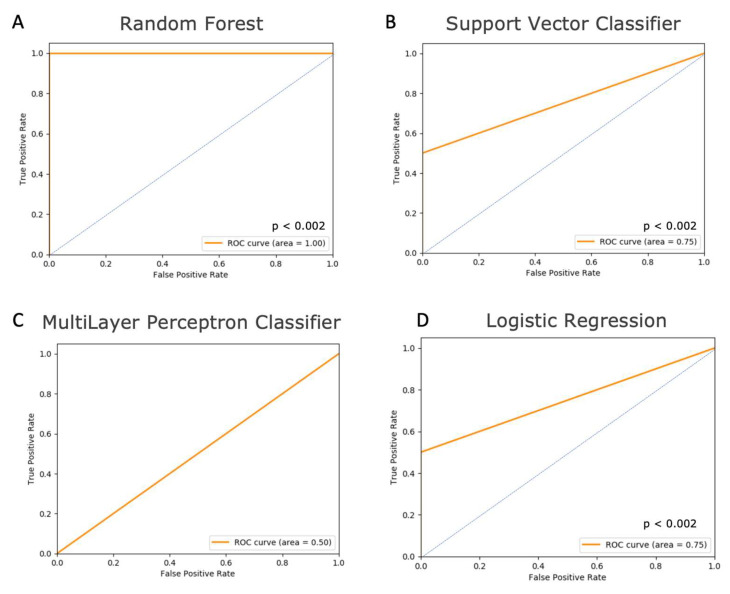
Receiver operating curves (ROC) for the three machine learning models assayed to predict distant metastases in the testing set of breast cancer patients from Duke University Hospital. (**A**) The random forest model yielded an area under the receiver operating curve (AUROC/AUC) of 1.0 for the testing dataset. (**B**) The ROC for the Support Vector Classifier yielded an AUC of 0.75 for the testing dataset. (**C**) The ROC for the multilayer perceptron classifier yielded an AUC of 0.5 for the testing dataset. No *p*-value was calculated as the model failed. (**D**) The ROC for the logistic regression model yielded an AUC of 0.75 for the testing dataset. Note: The blue line indicates the ROC curve of a random classifier.

The multimodal feature file consisting of the aforementioned clinicopathological features was set as “data”, while a binary column containing a feature file reporting disease recurrence at a distant site (i.e., distant recurrence flag; 0 for “no” and 1 for “yes”) for the respective patients (*n* = 161) was set as “label” for ImaGene. The model type was first set to RF in “Train” mode. Test size was set to “0.1 (i.e., 10% of dataset allocated to test)”, which partitioned the dataset into training (nTrain = 144) and testing (nTest = 17) sets. The K-fold cross-validation splitter parameter (i.e., ‘cv’) was set to “2”. Grid search was set to “True” to enable the execution of a grid search through the hyperparameters: max_depth = [6, 9, 10, 12, 15, 20] and cv = 4. The “data” normalization method was set to “StandScaler”, while the “label” normalization was set to “none” owing to its binary nature. Furthermore, the absolute correlation threshold parameter pre-ML-training was set to “−1.0” to silence the filtering of features based on Pearson’s correlation co-efficient threshold, thereby considering all the clinicopathological features for further training of the RF model. The run took approximately two minutes to yield a report on model performance (Supplementary Materials Report 1, Figure 2A, and Table 1).

Secondly, the model type was set to SVC and run with default parameters: cv = 2, test_size = 0.1, pre-ML correlation-threshold set to “−1.0”, data-normalization to “Stand Scaler”, and label-normalization to “none”. Furthermore, the grid_search was set to ‘True’ to perform a grid search for the SVC model through polynomial degree hyperparameters (i.e., kernel = [‘poyl’], degree = [3, 4, 5, 6, 7, 8, 9] and cv = 2; Supplementary Materials Report 2, Figure 2B, and Table 1).

Thirdly, a supervised neural network, the MLP classifier, was employed with default parameters. Subsequently, a grid search was executed setting test_size to 0.1 and using the following hyperparameters: Solver = [‘sgd’,‘bfgs’,‘adam’], alpha = [0.0001, 0.001, 0.01, 0.02, 0.05], hidden_layer_sizes = [(9,),(9,9,),(9,9,9,)] and cv = 6 (Supplementary Materials Report 3, Figure 2C, and Table 1).

Lastly, the model type was set to LogitR and run with both default model parameters and in grid search mode. To achieve optimal performance, the train:test ratio was modified to 85:15 followed by the grid search performed on the following hyperparameters: Solver: [‘liblinear’, ‘newton-cg’, ‘lbfgs’, ‘sag’, ‘saga’] and cv = 4 (Supplementary Materials Report 4, Figure 2D, and Table 1).

### 2.2. Validation Using ImaGene

**Figure 3:**
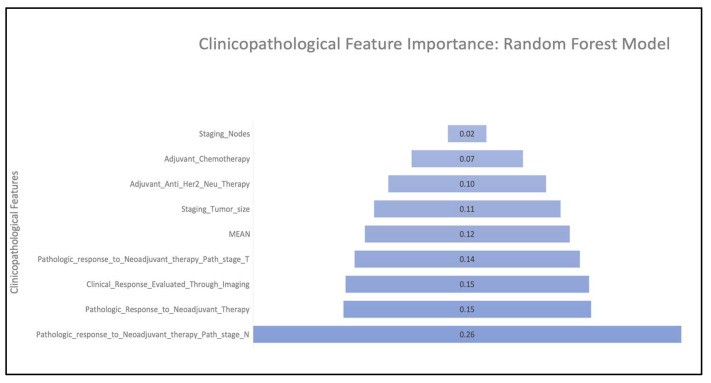
Feature importance scores of clinicopathological features from the random forest model used to predict distant recurrences in invasive breast carcinoma.

The model that performed best on the testing dataset from DHC was chosen for validation using the external IBC cohort at DHMC (Figure 1B). The validation study included a total of 67 IBC patients that had the same eight clinicopathological features collected from diagnosis to follow-up at DHMC. This validation dataset (i.e., nvalidate = 67) was screened for the following two inclusion criteria: (a) Neoadjuvant therapy administered and (b) onsite (i.e., DHMC-based) follow-up 24–45 months post adjuvant therapy. Of these 67 patients, only 15 matched the inclusion criteria. Out of these 15, only three patients exhibited distant recurrences, leading us to select eight patients at random (i.e., two with and six without distant recurrences) to balance the dataset and arrive at a more realistic validation AUC. The validation report from ImaGene showcased the performance of the validation dataset through the model (Supplementary Materials Report 5, Figure 3, and Table 2).

## 3. Results

### 3.1. Model Performance

The training (nTrain = 144: DUH) and testing (nTest = 17: DUH) of the models (i.e., RF, SVC, MLP, and LogitR) using ImaGene yielded a detailed performance report in html format, along with the supporting tables in csv format to facilitate the evaluation of these models (Supplementary Materials Reports 1–4, Figure 2, and Table 1). These reports and the supporting tabular output files showcased several metrics and plots collectively, which included cross-validation (CV) score, grid search CV score (when grid search is set to “True”), actual-vs-predicted-values scatter plots, and area under the receiver operating curve (AUROC or AUC), and the respective *p*-value for predicting a distant recurrence flag. Additionally, the Mean Square Error (MSE) and R-square (R2) from the model performance on the test dataset were also reported. The MSE, R2, and AUC metrics from all models facilitated the comparison of their performance through the test dataset.

Training the RF model yielded a best grid search CV score of 0.8 (Supplementary Materials Report 1), indicating the best score obtained from the 4-fold cross-validation performed during the tuning of the model through the aforementioned hyperparameters. Testing the RF model yielded MSE, R2, and AUC values of 0.0, 1.0, and 1.0 respectively, indicating a perfect prediction by the RF model (Supplementary Materials Report 1, Figure 2A, and Table 1). ImaGene ran permutations (i.e., shuffling) of labels (i.e., distant recurrence binary values) across test samples (*n* = 17) to infer the statistical significance of the prediction of the R2 and AUC reported by the model, indicating a strong prediction (i.e., *p* < 0.002).

Training the SVC model yielded a best grid search CV score of 0.78 (Supplementary Materials Report 2). Testing the SVC model yielded MSE, R2, and AUC values of 0.06, 0.43, and 0.75 respectively, indicating that the model performed considerably well (Supplementary Materials Report 2, Figure 2B, and Table 1). The performance of the SVC model was also deemed to be significant (*p* < 0.002); however, this model did not perform as well as the RF model, indicating the superiority of the ensemble of decision tree algorithms (i.e., RF) compared to support vectors for the classification of distant recurrence flags using the clinicopathological profiles of the IBC patients in the present study.

Training of the MLP model yielded a best grid search CV score of 0.83 (Supplementary Materials Report 3). However, testing this model yielded a very high MSE (i.e., 0.29), and AUC and R2 values of 0.5 and −0.41 respectively, indicating complete failure of the model (Supplementary Materials Report 3, Figure 2C, and Table 1). Furthermore, this demonstrates that supervised neural networks are not superior to RF and SVC methods for the classification of distant recurrences using clinicopathological profiles of IBC in the present study.

Lastly, training of the LogitR model yielded a best grid search CV core of 0.75 (Supplementary Materials Report 4). The testing of this model yielded MSE, R2, and AUC values of 0.08, 0.41, and 0.75 respectively, indicating that the model performed considerably well (Supplementary Materials Report 4, Figure 2D, and Table 1). The performance of the LogitR model was also deemed to be significant (*p* < 0.002). LogitR showed similar performance to SVC but lower compared to RF, reinstituting the superiority of ensemble decision trees (i.e., RF) compared to the logit method (i.e., LogitR) for the prediction of distant recurrence flags using clinicopathological profiles in IBC patients.

The RF model was the best-performing model in the present study (Figure 1B). The feature importance scores of clinicopathological features for predicting distant recurrences using the RF model indicate that the top four features exhibited scores greater than or equal to the mean of all scores (Figure 3). These features are (in order of importance) (1) pathologic response to neoadjuvant therapy evaluated using node status “N” (i.e., N0–N3), (2) pathologic response to neoadjuvant therapy (i.e., complete, incomplete, or DCIS-only remaining), (3) clinical response to neoadjuvant therapy as evaluated using SOC radiological images (i.e., complete response, incomplete response, or assessment unavailable), and (4) pathologic response to neoadjuvant therapy as evaluated using pathology stage “T” tumor size (T0–T4). The RF model was selected further for validation using the external cohort from DHMC (Figure 1B).

### 3.2. Model Validation

**Figure 4:**
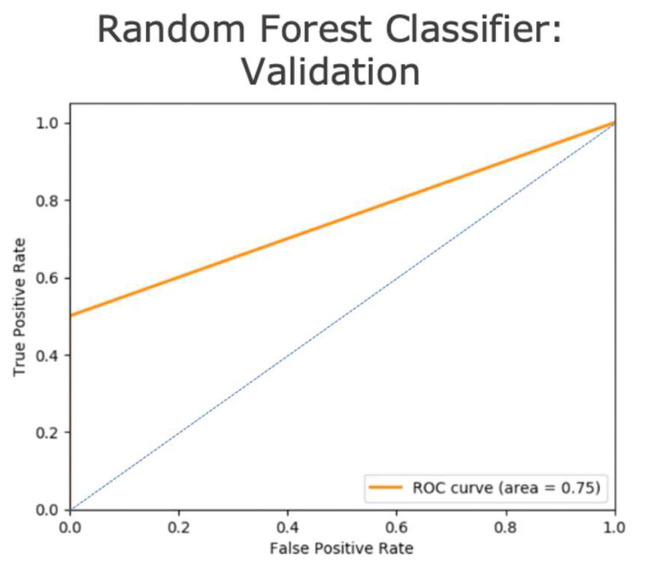
Receiver operating curve (ROC) for random forest model validated on the external Dartmouth Hitchcock Medical Center cohort, depicting an area under the curve (AUC) of 0.75. The AUC represents the validation of random forest model for the prediction of distant recurrence flags from the clinicopathological profiles in patients at the external site. Note: The blue line indicates the ROC curve of a random classifier.

The validation of the RF model was conducted on 8 out of 67 patients matching the inclusion criteria of having received therapy and followed up at DHMC 24–45 months after adjuvant therapy. The validation MSE of the model was found to be 0.125, with an AUC of 0.75 and an R2 of 0.33 (Supplementary Materials Report 5, Figure 4, and Table 2). This indicates that the RF model performed considerably well given the small size of the clinical dataset after screening for inclusion criteria as previously mentioned, highlighting a common issue in clinical datasets for lengthy studies that include clinicopathological results collected from the same site. Moreover, low AUC could also be attributed to interobserver variability in tumor and node evaluation pre- and post-therapy by clinicians and pathologists across two different hospital sites (i.e., DUH and DHMC).

## 4. Discussion

To the best of our knowledge, our study is the first ML/AI-based study to predict distant recurrences or metastasis in IBC patients using clinicopathological data collected from initial diagnosis through follow-up in the clinic and subsequently validated in an external dataset (i.e., cross-institutional). The random forest (RF) technique outperformed other models using the testing and validation sets across two hospital sites (i.e., DUH and DHMC). The key clinicopathological features impacting the prediction of metastasis are tumor responses to neoadjuvant therapy (NAT) evaluated using SOC imaging and pathology (i.e., Tumor (T) and Node (N) staging after neoadjuvant therapy) (Figure 4). The pathological evaluation of tumors and nodes post-neoadjuvant therapy is the gold standard in evaluating tumor response to neoadjuvant therapy [45] and is a recommendation of the 8th edition of the American Joint Committee of Cancer [46]. The use of SOC imaging is vital for the gross examination of tumors both before and after NAT (Viale and Fusco 2022) [46]. In our study, we found that the contribution of SOC imaging after NAT (i.e., for evaluating the tumor response to NAT) was more impactful than imaging before NAT in the prediction of metastasis (Figure 4). In contrast, we found that the binary status of adjuvant therapy did not make a substantial contribution to the prediction of metastases (Figure 4). This is in agreement with previous studies in an international breast cancer cohort, in which the adjuvant therapy could result in increased metastasis to the liver and central nervous system but decreased metastasis to bones [47,48,49], which supports the reduced contribution of adjuvant therapy to predict metastasis in our study as well (Figure 4).

Several ML/AI studies have been conducted in oncology [29,30,31,32,33,34,35,50,51]. Some of these have attempted to predict 5–10-year breast cancer recurrences using both structured and unstructured clinicopathological data from Electronic Health Records (EHRs) [50,51]; however, these studies focused primarily on predicting local rather than distant recurrences. A previous study [52] predicted distant recurrences in breast cancer from both unstructured and structured clinical data in EHR using natural language processing and deep learning algorithms but lacked structured clinicopathological predictors such as those used in our study. Furthermore, the same study [52] also lacked advanced feature engineering. In contrast, the present study used structured clinicopathological data from EHRs that were further curated by trained pathologists and medical residents at the hospital prior to being fed to the ML model. Moreover, the present study provides us with an advanced understanding of the contribution of each clinicopathological feature towards the prediction of distant recurrence in the form of feature importance scores (which measures the weights of the features used to predict the label, i.e., risk of distant recurrence), which could guide feature engineering efforts in the future (Figure 3). Additionally, several previous ML studies have predicted disease recurrences in a variety of other cancers, such as in non-metastatic renal cell carcinoma [53] and in early-stage endometrial cancer [54] which only predicted recurrences at an AUC of 0.53 owing to the small size of the dataset. In contrast, the present study achieves AUC values of 1.0 and 0.75 in the intra- and inter-institutional tests respectively, despite the small sample size (i.e., ntest = 17 and nvalidate = 8), highlighting the importance of highly curated data in ML studies.

Although the use of ML in predictive models can increase our ability to predict outcomes in individual cancer patients, validation is vital to the incorporation of ML into clinical workflows (Kourou 2015) [55]. A previous study that predicted distant recurrences in breast cancer used both classification-based and deep learning models but repurposed approximately 10% of their training data for validation [52]. Another study that predicted distant recurrence in breast cancer using clinicopathological and serum HER2 profiles yielded an AUC of 0.8 for the testing set but lacked validation in an external cohort, calling its clinical utility into question [38]. Thus, although the present study was based solely on classification models, it did use a blinded clinical validation set from a completely different hospital site than the sites from which training and test originated, thereby highlighting the strength of the study and predictions.

However, the present study has several limitations. Notably, the number of samples in the testing and validation sets was relatively low. In the near future, we plan to enhance the testing and validation datasets by collating data from multiple institutions to encompass a wider range of heterogenous IBC tumors and institutions. This could be performed using an open-source platform such as ImaGene, which enables the democratization of multi-omic analyses and gives open access to results [41]. Another limitation of the present study is the lack of information regarding the site of distant recurrence, which was not provided by the dataset shared by DUH via the TCIA platform. With this additional information, the present study could be used as a blueprint for predicting the site of distant recurrence as well.

Using the RF model to predict the possibility of distant recurrences in breast cancer patients could provide clinicians with the ability to foresee distant recurrences and tailor treatment and management plans accordingly to improve outcomes. Furthermore, our study extends the capability of ImaGene to utilize several clinicopathological features of each patient’s tumor throughout the diagnostic and therapeutic journey. Using ImaGene, a patient’s unique pathologic, radiologic, and therapeutic information can be leveraged to predict distant recurrences using various ML/AI models in IBC. ImaGene is an open-access software that shares the code for the automated operation of ML/AI models, supporting the repeatability of their training, testing, and validation with datasets at any institution worldwide, unlike the code used by previous studies that sought to predict distant recurrences using clinicopathological features of cancer [36,37,38]. Our study also proves the cross-validity of the RF model across two distinct hospital sites. A similar model could potentially be trained, tested, and validated for the prediction of disease progression in other cancer types based on the clinicopathological profiles of the respective tumors in the future. Our study advances the field of non-invasive predictions of cancer metastasis. Future research in this field could aid researchers and clinicians in identifying the risk and sites of disease recurrence, thereby optimizing cancer treatment and ultimately reducing cancer mortality.

## 5. Conclusions

This study explores ML models for predicting metastasis risk in IBC patients using clinicopathological features of their tumor and lymph nodes measured pre- and post-neoadjuvant therapy, including adjuvant therapy status. Classification-based ML models were trained and tested on one hospital’s (DUH) datasets. The best model (RF) was further validated using another hospital’s (DHMC) dataset, demonstrating significant AUC and R^2^ values for cross-validity in heterogenous IBC tumors sitewide. Tumor response to neoadjuvant therapy, evaluated through SOC imaging and pathology (including the tumor and node staging), contributed most to metastasis prediction. ML models hold the potential for stratifying patients into high- and low-risk categories for metastasis, enabling the regulation of surveillance and treatments to improve their quality of life.

## Figures and Tables

**Table 1:** Results for various classifiers for the test dataset at DUH.

Models	AUC	R^2^	RMSE:Stdev
Random Forest	1.0	1.0	0.0
Support Vector Classifier	0.75	0.43	0.75
Multilayer Perceptron Classifier	0.5	−0.41	1.19
Logistic Regression	0.75	0.4	0.77

**Table 2:** Validation of the random forest model at external site: DHMC.

Model	AUC	R^2^	RMSE:Stdev
Random Forest	0.75	0.33	0.82

## Data Availability

Data are available at: https://github.com/skr1/Imagene (accessed on 1 January 2023).

## Supplementary Materials

The following supporting information can be downloaded at: https://github.com/skr1/Imagene. Reference [56] are cited in the supplementary materials.
